# Effect of Freeze Drying and Hot Air Drying on the Composition and Bioactivities of Lipids from Razor Clam *Sinonovacula constricta*

**DOI:** 10.3390/foods14060915

**Published:** 2025-03-07

**Authors:** Dexu Wang, Runjia Chang, Changyu Liu, Jiaxun Li, Jibin Liu, Ning Li, Yun Zhang, Xiaobin Li, Peihai Li, Kechun Liu

**Affiliations:** 1 Engineering Research Center of Zebrafish Models for Human Diseases and Drug Screening of Shandong Province, Biology Institute, Qilu University of Technology (Shandong Academy of Sciences), Jinan 250103, China; dexuwangbio@163.com (D.W.); liuchangyu000422@163.com (C.L.); 18287319631@163.com (J.L.); liujibin310@163.com (J.L.); lining@sdas.org (N.L.); xiaohan_0818@163.com (Y.Z.); lixb@sdas.org (X.L.); 2 School of Life Sciences and Technology, Tongji University, Shanghai 200092, China; 2152673@tongji.edu.cn

**Keywords:** UPLC-MS/MS-based lipidomics, zebrafish model, biological activity, drying method, shellfish lipids

## Abstract

Razor clams, which are rich in diverse lipids, are notable for their unique health benefits and functional properties. This study comprehensively characterized and compared the composition and bioactivities of razor clam lipids after freeze drying (FD) and hot air drying (HD) using UPLC-MS/MS-based lipidomics and zebrafish models. Lipidomics analysis identified 1056 lipids classified into five lipid classes, among which glycerophospholipid (GP) was the most abundant, accounting for 57.39% of the total lipids. The total lipids were also grouped into 24 lipid subclasses, including dominated triglycerides, phosphatidylethanolamines, and phosphatidylcholines. Differential lipid species were identified between the FD, HD, and fresh (FS) sample groups, with 174, 141, and 154 species differing between FD vs. FS, HD vs. FS, and FD vs. HD, respectively. The antithrombotic, anti-inflammatory, and antioxidant activities of lipids extracted from FD, HD, and FS razor clams were evaluated using the zebrafish model. Lipids from FD and FS razor clams exhibited all bioactivities at some concentrations, while HD lipids showed antithrombotic and anti-inflammatory activities but lacked antioxidant activity. In summary, the lipid composition and bioactivities of fresh razor clams were altered following FD and HD processes, with significant differences observed between the two methods. These findings underscore the nutritional value of fresh razor clams after processing and provide insights for developing razor clam products.

## 1. Introduction

*Sinonovacula constricta*, commonly known as the razor clam, is an elongated bivalve naturally distributed along the west coast of the Pacific Ocean [1]. In China, razor clams are highly valued as a seafood product for their delicious flavor and nutritional richness [2]. Shellfish lipids, compared to those from terrestrial plants and animals, exhibit a complex fatty acid composition with a high content of omega-3 long-chain polyunsaturated fatty acids (n-3 LC-PUFAs), particularly eicosatetraenoic acid (EPA) and docosahexaenoic acid (DHA), which are effective in managing cardiovascular diseases [3]. Razor clams contain substantial amounts of EPA and DHA, each comprising approximately 10% of the total fatty acids [4], making them an excellent source of LC-PUFA to meet human nutritional requirements. Recently, n-3 LC-PUFAs in their phospholipid (PL) form have garnered significant attention due to their superior bioavailability, enhanced tissue-delivery capacity, and better health-promoting effects [5]. Razor clam meat is tender, flavorful, and nutrient-rich, offering certain medicinal benefits. It is not only high in protein, lipids, total sugars, amino acids, and monosaccharides, but also contains abundant trace elements such as iodine, selenium, zinc, and manganese, contributing to brain health, detoxification, and antioxidant activity [6]. Consequently, razor clams can be regarded as a “health” food. The annual production of razor clams in China is about 800,000 tons, accounting for about 60% of the world’s production. At present, the consumption market for razor clams shows a stable growth trend, and razor clams are mainly consumed in the forms of fresh and processed food [7]. With the improvement of people’s living standards and the pursuit of healthy food, razor clams are becoming more and more popular with consumers around the world.

With the advancement of razor clam culture technology, cultivating high-quality varieties offers advantages such as a short growth cycle, low cost, and high economic returns [8]. Consequently, razor clams hold significant potential for further development and utilization. However, the high water content and nutrient richness of fresh aquatic products make them prone to microbial growth [9]. Additionally, the lipids in aquatic products may undergo complex reactions and transformations during storage due to factors such as lipid composition and storage and transportation conditions, leading to changes in lipid molecules that can affect product quality [10]. Razor clam production is generally confined to coastal regions, where the primary sales are fresh. In inland areas, sales are limited to canned, dried, or frozen products [11]. Therefore, timely preservation and processing are essential. Drying is a key processing method to extend the shelf-life of aquatic products [12]. Dried products are favored by consumers for their nutritional richness and ease of transportation. Drying methods, including sun drying, hot air drying, freeze drying, microwave drying, and mixed drying, are widely employed to reduce moisture content and enhance the shelf-life of seafood [13]. Different drying processes may affect lipid profiles by lipid oxidation and hydrolases, which is associated with the drying method, temperature, and time [14,15]. The variability in lipid profiles obtained through different drying methods has attracted significant interest, particularly regarding differences in lipid composition and bioactivities under various drying conditions. However, limited research has been conducted on the effects of drying techniques on the lipid composition and bioactivities of razor clams.

Dried razor clams are a common shellfish commodity, but the molecular and bioactivity changes during the drying process remain unclear. This study aimed to comprehensively characterize and compare the lipid composition and bioactivities of razor clams subjected to freeze drying (FD) or hot air drying (HD) for the first time. Lipid samples were extracted separately from fresh (FS), FD, and HD razor clams and analyzed using UPLC-MS/MS-based lipidomics. Additionally, the antithrombotic, anti-inflammatory, and antioxidant activities of all lipid samples were evaluated using in vivo zebrafish models. These findings enhance our understanding of the lipid composition and bioactivities of razor clams following FD or HD processes and support the development of razor clam products.

## 2. Materials and Methods

### 2.1. Chemicals and Regents

Fresh razor clams were purchased from a local market in Jinan, Shandong, China. All chemicals and solvents used for sample extraction and preparation were of an analytical grade. Arachidonic acid (AA) was obtained from Stanford Analytical Chemicals Co., Ltd. (Stanford, CA, USA). Aspirin (ASP) was sourced from Beijing Spectrum Analysis Standard Technology Co., Ltd. (Beijing, China). Methylene blue, dimethyl sulfoxide (DMSO), anhydrous sodium acetate, anhydrous ethanol, hydrogen peroxide, ammonium acetate, and CuSO_4_ were purchased from Sinopharm Chemical Reagent Co., Ltd. (Shanghai, China). 1-Phenyl-2-thiourea (PTU) and o-dianisidine were obtained from Sigma Co., Ltd. (St. Louis, MO, USA). Metronidazole and vitamin C (Vc) were sourced from Macklin Co., Ltd. (Shanghai, China). Methyl cellulose, indomethacin, cholic acid-D4, and D-insect fluorescein free acid (mixed internal standard) were purchased from Yuan Ye Biotechnology Co., Ltd. (Shanghai China). Pure water was supplied by Wahaha Group Co., Ltd. (Hangzhou China), while deionized water and streptavidin E were purchased from Solarbio Co., Ltd. (Beijing, China). Paraformaldehyde (4%) was obtained from White Shark Biotechnology Co., Ltd. (Hefei, China). Methanol, acetonitrile, and isopropanol were sourced from Thermo Fisher Scientific Co., Ltd. (Waltham, MA, USA), and chloroform was purchased from Greagent Co., Ltd. (Shanghai, China). All chemicals and reagents used in this study were of an analytical grade.

### 2.2. Drying and Extraction of Lipid Samples

The edible parts (1650 g) of razor clams were collected and randomly divided into three groups of equal weight. One group was freeze-dried using a freeze dryer (Scientz Biotechnology Co., Ltd., Ningbo, China) for 24 h under a vacuum at a low temperature. Another group was dried in hot air at 70 °C using an electric drying oven (Jinghong Experimental Equipment Co., Ltd., Shanghai, China) for 24 h [16]. The remaining group served as the control, representing the initial, unprocessed sample. The drying processes adhered to the Chinese National Standard for Aquatic Processing [17], ensuring that moisture content was reduced to below 22%. Each drying method yielded 70 g of dried sample. Lipids were extracted from the hot air-dried, freeze-dried, and fresh razor clam meat samples using a previously established method [18]. After extraction, the lipid samples were cut into small pieces and then subjected to crushing and homogenization using an automatic rapid sample grinder (Wanbai Wonbio-E, 200 W, Shanghai, China). Subsequently, the homogenized tissue fluid was immersed in 1 L of 95% ethanol. Three rounds of extraction were carried out using a continuous ultrasonic cleaner (Scientz SB-5200DT, 360 W, Ningbo, China), with each extraction lasting for 8 h. Then, the supernatant was collected and concentrated for the subsequent activity experiments.

### 2.3. Lipid Pre-Treatment

All lipid samples were prepared and analyzed by OE Biotech, Inc. (Shanghai, China). Each lipid sample (30 mg) was weighed, and 600 μL of isopropanol-methanol (1:1, *v*/*v*) along with two small steel balls were added. The samples were pre-cooled at −20 °C for 2 min and then ground using a grinder (60 Hz, 2 min). The samples were extracted by pulsed ultrasonication (Fuyang Technology F-060SD, 480 W, Shenzhen, China) for 10 min, allowed to stand at −20 °C for 20 min, and centrifuged at 13,000 rpm for 10 min at 4 °C. The supernatants were transferred into Ultra-High Performance Liquid Chromatography-MS/MS (UPLC-MS/MS) injection vials with inner lining pipes for UPLC-MS/MS analysis. Quality control (QC) samples were prepared by mixing equal volumes of extracts from all samples, ensuring that the QC volume matched the sample volume. All extraction reagents were pre-cooled to −20 °C before use. Four parallel studies were conducted for each group of samples.

### 2.4. Lipid Composition Analysis

Lipid composition was analyzed using a UPLC-MS/MS system (ACQUITY UPLC I-Class Plus connected with a Q Exactive high-resolution mass spectrometer). The column (ACQUITY UPLC BEH C8, 100 mm × 2.1 mm, 1.7 μm, Waters Co., Ltd., Milford, MA, USA) temperature was maintained at 55 °C, with a flow rate of 0.26 mL/min and an injection volume of 3 μL. Mobile phase A consisted of acetonitrile:pure water (6:4, *v*/*v*), containing a final concentration of 10 mM of ammonium acetate in the mixture, while mobile phase B was isopropanol:acetonitrile (9:1, *v*/*v*), also containing a final concentration of 10 mM of ammonium acetate in the mixture. The elution gradient was as follows: 32% B from 0 to 1.5 min, a linear increase to 85% B from 1.6 to 15.5 min, 97% B from 15.6 to 18 min, and 32% B from 18.1 to 20 min. The experimental procedures included pre-treatment and sample on-boarding, and the evaluation of the stability of the mass spectrometry system through quality control using internal standards (4 μg/mL, methanol configuration) and QC samples. Subsequently, the raw data in a raw format exported by Q Exactive LC-MS/MS were analyzed by Lipid Search software, which then read the exact mass numbers of MSn and parent ions. Based on the parent ions and multistage mass spectrometry data in each individual sample, the software identified the structures of lipid molecules and their positive and negative ion addition patterns using the LipidSearch version 5.0 database. More details can be found in the Supplementary Materials.

### 2.5. Zebrafish Maintenance and Larvae Collection

The zebrafish strains used in this study included wild-type AB and transgenic lines *Tg (Krt4-NTR:GFP)* and *Tg (zlyz:EGFP)*. Zebrafish were maintained following the NIH Guide for the Care and Use of Laboratory Animals (No. 8023, amended in 1996). The fish were kept under a 14-h light/10-h dark cycle at 28 ± 0.5 °C and fed artemia twice daily. Mature zebrafish were placed in breeding tanks at a male-to-female ratio of 2:2, with the baffle plate removed the next morning to facilitate mating. Embryos were collected and maintained in culture water containing 5 mM of NaCl, 0.17 mM of KCl, 0.33 mM of CaCl_2_, and 0.33 mM of MgSO_4_ supplemented with methylene blue solution (0.5 mg/L). After incubation in a temperature-controlled light incubator at 28 ± 0.5 °C for 6 h, dead embryos were removed promptly, and the embryo culture water was replaced every 24 h. All animal experiments and protocols complied with the guidelines of the Animal Care and Ethics Committee of the Biology Institute, Qilu University of Technology (Shandong Academy of Sciences).

### 2.6. Antithrombotic Assay

The antithrombotic activity of lipids was evaluated in AB zebrafish larvae at 72 h post-fertilization (hpf) using a modified version of a previously described method [19]. Healthy zebrafish larvae were selected under a stereomicroscope, randomly transferred into 24-well plates (10 larvae in 2 mL of culture water per well), and divided into five groups: a control group, a model group, a positive control group, and three sample groups representing different concentrations of lipid extracts. The model group was treated with 80 μM of AA. The positive control group received 80 μM of AA and 22.5 μg/mL of ASP. The sample groups were treated with 80 μM of AA and lipid extracts at three concentrations (20, 40, and 80 μg/mL), respectively. ASP and lipid extracts were added first to the positive control and sample groups, respectively, and incubated at 28 ± 0.5 °C for 6 h. Subsequently, AA was added to all groups except the control. After a 1.5 h incubation, zebrafish larvae were stained with 1 mg/mL of o-Dianisidine staining solution for 20 min at room temperature in the dark. The larvae were then washed three times with culture water, fixed with 4% paraformaldehyde, and positioned laterally on methylcellulose-coated slides. Heart images of the zebrafish were captured using a fluorescence microscope (Zeiss AXIO-V16, Baden-Württemberg, Germany). The experiment was conducted in triplicate. The area and intensity of heart erythrocyte staining, indicative of the number of cardiac erythrocytes, were quantified using Image-Pro Plus 5.1 software.

### 2.7. Anti-Inflammatory Assay

The anti-inflammatory activity of lipids was evaluated using *Tg (zlyz: EGFP)* zebrafish larvae at 72 h post-fertilization (hpf) with a modified version of a previously described method [20]. Healthy zebrafish larvae were selected under a stereomicroscope, randomly transferred into 24-well plates (10 larvae in 2 mL of culture water per well), and divided into five groups: a control group, a model group, a positive control group, and three sample groups corresponding to different concentrations of lipid extracts. The model group was treated with 40 μM of CuSO_4_. The positive control group received 40 μM of CuSO_4_ and 20 μM of indomethacin. The sample groups were treated with 40 μM of CuSO_4_ and lipid extracts at three concentrations (20, 40, and 80 μg/mL), respectively. Indomethacin and lipid extracts were added first to the positive control and sample groups, respectively, and incubated at 28 ± 0.5 °C for 2 h. CuSO_4_ was subsequently added to all groups except the control. After a 1 h incubation, zebrafish larvae were fixed with 4% paraformaldehyde and positioned laterally on methylcellulose-coated slides. Fluorescence-labeled immune cells undergoing migration were visualized and captured using a fluorescence microscope (Olympus SZX16, Hachioji, Japan). The experiment was conducted in triplicate. Migrating immune cells were quantified using Image-Pro Plus 5.1 software.

### 2.8. Antioxidant Assay

The antioxidant activity of lipids was evaluated using *Tg (Krt4-NTR: GFP)* zebrafish larvae at 24 h post-fertilization with a modified version of a previously described method [21]. Healthy zebrafish larvae were selected under a somatic microscope and incubated in a 1 mg/mL of streptavidin E solution for approximately 3 min to remove the outer layer of the egg membrane. Afterward, the healthy larvae were transferred into 24-well plates (10 larvae in 2 mL of culture water per well) and divided into five groups: a control group, a model group, a positive control group, and three sample groups corresponding to different lipid extract concentrations. The model group was treated with 5 mM of metronidazole, the positive control group was treated with 5 mM of metronidazole and 120 μg/mL of Vc, and the sample groups were treated with 5 mM of metronidazole and three concentrations of lipid extracts (20, 40, and 80 μg/mL), respectively. The zebrafish larvae in each group were placed in a thermostatic incubator at 28 ± 0.5 °C. After 24 h, the larvae were fixed with 4% paraformaldehyde and placed laterally on methylcellulose-coated slides. Fluorescent dots on the skin were visualized and captured using an inverted fluorescence microscope (Carl Zeiss, Axio Zoom.V16, Baden-Württemberg, Germany). The experiment was repeated three times, and the number of fluorescent dots was quantified using Image-Pro Plus 5.1 software.

### 2.9. Statistical Analysis

Lipid data were processed using Lipid Search software to read raw data exported by Q Exactive LC-MS/MS. The precise mass numbers of MSn and parent ions were obtained, and the structure of the lipid molecules was identified based on parent ions and multilevel mass spectrometry data from each independent sample. Multivariate statistical analyses were performed using SIMCA-P 14.1 (Umetrics AB, Umeå, Sweden), including unsupervised principal component analysis (PCA), supervised partial least squares discriminant analysis (PLS-DA), and orthogonal partial least squares discriminant analysis (OPLS-DA). The GraphPad Prism v.8.0 (GraphPad Software, La Jolla, CA, USA) was used for data analysis, and *t*-tests were conducted to assess significant differences between groups. All experimental data are presented as mean ± SEM. Statistical significance was defined as *p* < 0.05, and highly significant differences were defined as *p* < 0.01.

## 3. Results

### 3.1. Lipidomic Analysis

**Figure 1:**
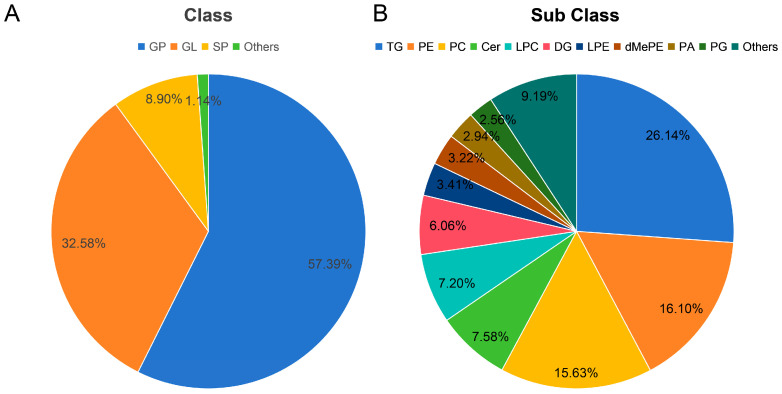
Lipid profiles in razor clams. (**A**) Lipid classes. (**B**) Lipid subclasses.

The lipid compositions in the HD, FD, and FS samples were analyzed using UPLC-MS/MS-based lipidomics (Figure 1, Table S1). A total of 1056 lipid types were identified and classified into five lipid categories: glycerophospholipid (GP), glyceride (GL), sphingolipid (SP), fatty acyl (FA), and Saccharolipid (SL). Among these categories, GP was the most abundant lipid class in razor clams, accounting for 57.39%. And, GP was further divided into 15 subclasses, primarily including 170 phosphatidylethanolamines (PEs), 165 phosphatidylcholines (PCs), and 76 lysophosphatidylcholines (LPCs). GL accounted for 32.58% of total lipids, and included 276 triglycerides (TGs), 64 diacylglycerols (DGs) and 4 monoglycerides (MGs). The total lipids were also grouped into 24 lipid subclasses, among which TGs, PEs, and PCs were predominant, accounting for 26.14%, 16.10%, and 15.63% of the total lipids, respectively. The molecular species of LPC (16:0e), PC (18:0/16:1), PE (18:0p/22:2), LPC (22:6), and PE (20:1p/19:1) were identified as the most abundant species (Table S1). Razor clam lipids contained a high proportion of polyunsaturated fatty acids (PUFAs), comprising 51.33% of the total lipids, including EPA, DHA, C18:3n-3, arachidonic acid (AA), and docosapentaenoic acid (DPA). GP also contained a high proportion of PUFAs (44.65% of GPs). Additionally, PUFA-TGs were the dominant components within the TG species, accounting for 70.84% of total TGs, PUFA-PEs accounted for 58.70% of total PEs, and PUFA-PCs accounted for 53.34% of total PCs.

### 3.2. Comparative Analysis of Differences in Lipids

Unsupervised principal component analysis (PCA) was performed to observe the overall distribution of samples and the stability of the entire analysis process [22]. In the PCA scatter plots (Figure S1), the FD, HD, FS, and quality control (QC) samples were separated into four distinct clusters, with the samples from the same group clustered together within the same ellipse, corresponding to a 95% confidence interval. This indicates that the lipid composition of the three groups differed significantly, and that the instrument platform detection was accurate and stable. Supervised partial least squares analysis (PLS-DA) was used to distinguish the differences in lipid profiles between groups. The PLS-DA scatter plots confirmed similar results, and the relevant parameters of the model indicated that it was valid and reliable.

**Figure 2:**
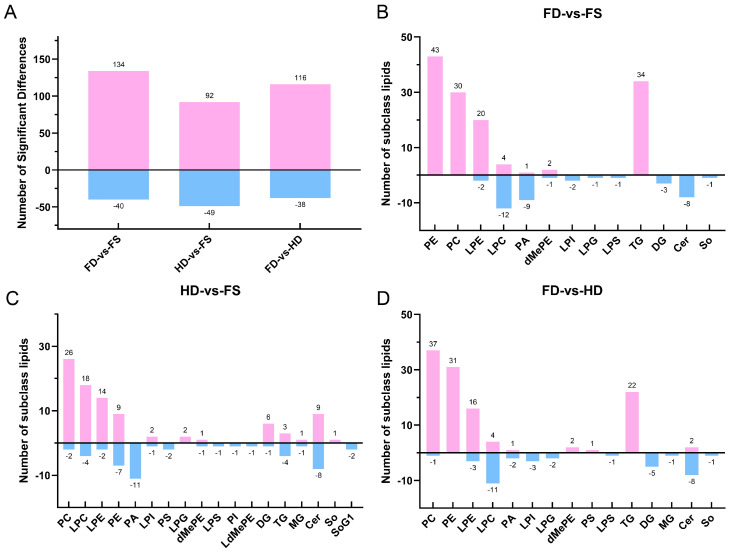
(**A**) The total number of different lipids in each comparison group. (**B**–**D**) The number of different lipid subclasses between the FD and FS, HD and FS, FD and HD groups. FD-vs-FS compares FD and FS. HD-vs-FS compares HD and FS. FD-vs-HD compares FD and HD. Pink represents a higher level and blue represents a lower level.

Using a variable importance in the projection (VIP) > 1.0 and a *p* value < 0.05 from the PLS-DA model and statistical analysis, differential lipids among the FD, HD, and FS groups were identified (Figure 2). A total of 174 differential lipid species were found between the FD and FS groups, including 13 lipid subclasses, such as 43 PEs, 34 TGs, 30 PCs, 22 lysophosphatidylethanolamines (LPEs), and 16 LPCs. There were 141 differential lipid species between the HD and FS groups, including 18 lipid subclasses, such as 28 PCs, 22 LPCs, 17 Ceramides (Cers), 16 Pes, and 16 LPEs. Compared to the HD group, the FD group exhibited significant differences in 154 lipid species, including 15 lipid subclasses, such as 38 PEs, 31 PCs, 22 TGs, 19 LPEs, and 15 LPCs.

The volcano plot and lollipop map were analyzed to provide an intuitive display of the differences in lipid composition between FD, HD, and FS samples based on the identified lipid profiles (Figures S2 and S3, Tables S2–S4). Compared to FS samples, the FD samples exhibited 134 differential lipids with higher levels, including TG (16:1/16:1/18:3), PE (14:0/20:5), TG (16:1/14:1/18:3), and LPE (22:3), among others. Additionally, 40 differential lipids were found at lower levels, including Cer (d20:2/2:0), LPI (18:0), Cer (d20:1/2:0), and LPI (20:1). In contrast, the HD samples had 92 differential lipids with higher contents, including Cer (d17:1/2:0), Cer (d20:1/4:0), Cer (d18:2/2:0), and Cer (d17:1/4:0). The HD samples also had 49 differential lipids with lower contents, including SoG1 (d18:1), SoG1 (d16:1), PI (26:6/18:0), and PC (16:1/22:6). Compared to the HD samples, the FD samples displayed 116 differential lipids at higher levels, including PC (17:1/22:6), PC (16:1/14:0), PE (14:0/20:5), and PC (16:1/22:6). Additionally, 38 differential lipids were found at lower levels, including Cer (d17:1/4:0), Cer (d20:1/4:0), Cer (d20:2/2:0), and Cer (d20:1/2:0).

### 3.3. Evaluation of the Antithrombotic Activity of Lipids

**Figure 3:**
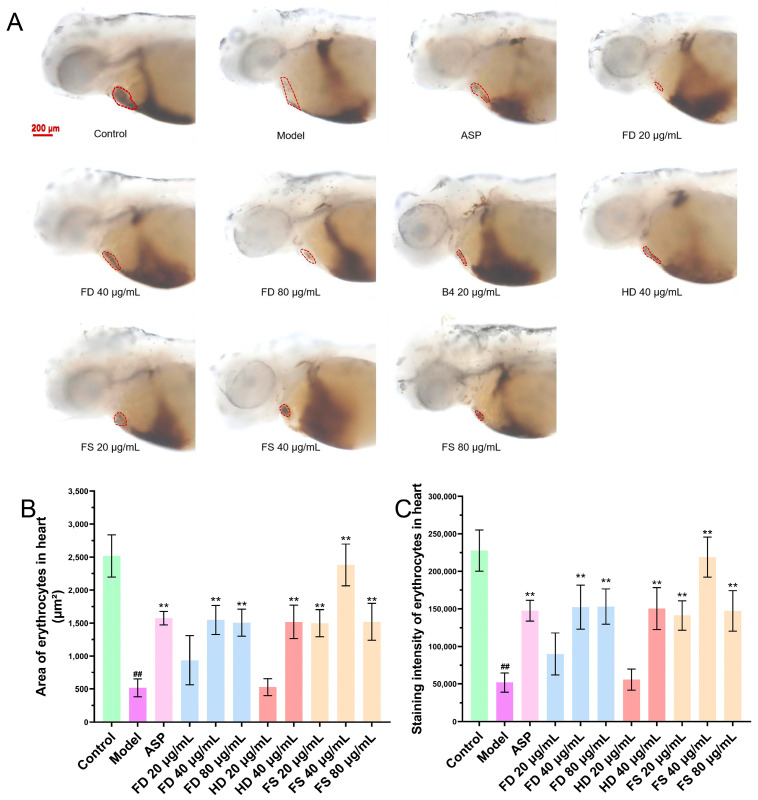
The antithrombotic activity of lipids. (**A**) Representative images of heart erythrocyte staining (scale bar: 200 μm), indicated with the red dotted circle. (**B**) Quantitative analysis of the heart erythrocyte staining area in the zebrafish (*n* = 10, mean ± SEM). (**C**) Quantitative analysis of heart erythrocyte staining intensity in the zebrafish (*n* = 10, mean ± SEM). Compared to control group, ^##^ *p* < 0.01; compared to model group, ** *p* < 0.01.

The formation of thrombus can reduce the return blood volume in zebrafish, as evidenced by a reduction in the heart erythrocyte area and a decrease in staining intensity [19]. To assess this, we examined the heart erythrocyte area and staining intensity in zebrafish. The effects of each sample group on the heart erythrocyte area and staining intensity are shown in Figure 3. The results indicated that the staining area and intensity of heart erythrocytes in the model group were significantly reduced compared to the control group, suggesting thrombus formation and a reduction in the return blood volume in the model group zebrafish. Compared with the model group, the 40 and 80 μg/mL lipid samples from the FD group, 40 μg/mL lipid samples from the HD group, and 20, 40, and 80 μg/mL lipid samples from the FS group showed a significant increase (*p* < 0.01) in the staining area and intensity of zebrafish heart erythrocytes, which was consistent with the results of the positive control group. These findings suggest that FD, HD, and FS lipids exhibit antithrombotic activity at certain concentrations. Furthermore, the FS lipid group demonstrated superior antithrombotic activity compared to the other two groups at a concentration of 40 μg/mL. At the concentration of 80 μg/mL, zebrafish in the HD group died, so statistical analysis could not be performed for this group.

### 3.4. Evaluation of Anti-Inflammatory Activity of Lipids

**Figure 4:**
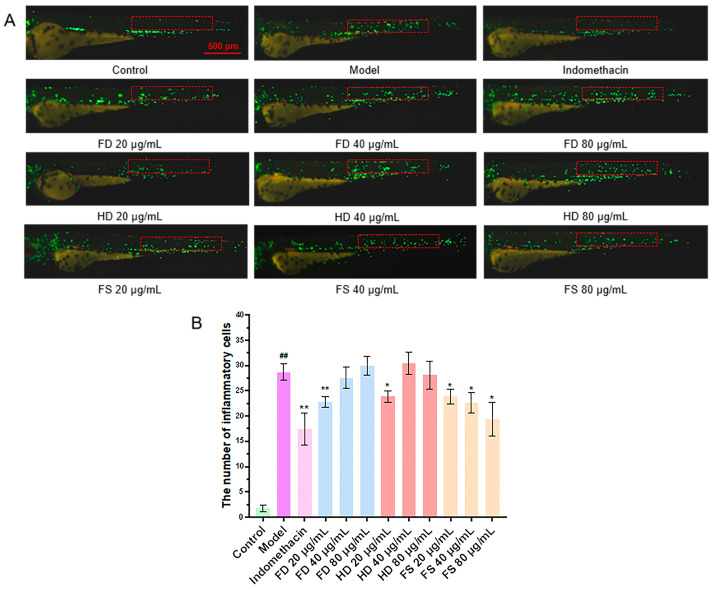
The anti-inflammatory activity of lipids. (**A**) Representative images of the inflammatory response in zebrafish, with the red box indicating the statistical area of inflammatory cells (scale bar: 500 μm). (**B**) Quantitative analysis of the number of migrated inflammatory cells in the zebrafish (*n* = 10, mean ± SEM). Compared to the control group, ^##^ *p* < 0.01; compared to the model group, * *p* < 0.05, ** *p* < 0.01.

It has been demonstrated that CuSO_4_ can induce a robust acute inflammatory response in zebrafish, triggering the migration of immune cells to the vicinity of lateral line neuroblasts [20]. The reduction in the number of migrating immune cells can be used to evaluate the anti-inflammatory effect. The results showed that the number of fluorescently labeled immune cells migrating to the lateral line was significantly higher in the CuSO_4_ model group compared to the control group (Figure 4), confirming the successful induction of the zebrafish inflammation model by CuSO_4_. The number of migrating immune cells in the FD, HD, and FS groups significantly decreased (*p* < 0.05) at a concentration of 20 μg/mL compared to the model group. Furthermore, the number of immune cells in the FS group also decreased (*p* < 0.05) at concentrations of 40 and 80 μg/mL. These findings suggest that the FD (20 μg/mL), HD (20 μg/mL), and FS (20, 40, and 80 μg/mL) lipid groups exhibit anti-inflammatory activity. The anti-inflammatory effect of the FS lipids was more pronounced than that of the other two groups at concentrations of 40 and 80 μg/mL.

### 3.5. Evaluation of Antioxidant Activity of Lipids

**Figure 5:**
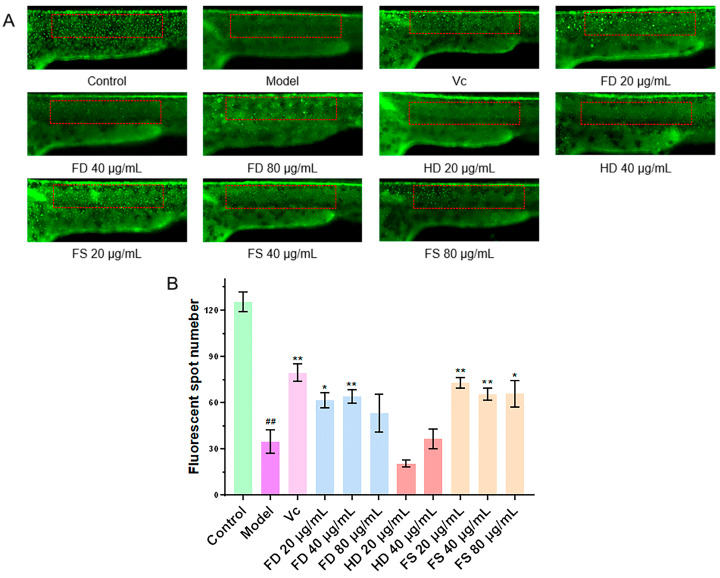
The antioxidant activity of lipids. (**A**) Representative images of transgenic zebrafish skin fluorescent dots, with statistical areas of fluorescent dots in red boxes (scale bar: 500 μm). (**B**) Quantitative analysis of transgenic zebrafish skin fluorescent dots (*n* = 10, mean ± SEM). Compared to the control group, ^##^ *p* < 0.01. Compared to the model group, * *p* < 0.05, ** *p* < 0.01.

Metronidazole induced the excessive production of reactive oxygen species, leading to the apoptosis of skin cells and a reduction in skin fluorescent spots [21]. The antioxidant capacity of the lipid samples was evaluated by detecting the number of fluorescent dots on the zebrafish skin. Compared to the control group (Figure 5), the number of fluorescent dots was significantly reduced in the model group, indicating that metronidazole caused oxidative damage in zebrafish and a decrease in fluorescent dots. The FS lipids (in amounts of 20, 40, and 80 μg/mL) and FD lipids (in amounts of 20 and 40 μg/mL) exhibited significantly more fluorescent dots than the model group (*p* < 0.05), indicating that these concentrations of FS and FD lipids possess significant antioxidant activities. However, the HD lipids (20 and 40 μg/mL) did not show significant antioxidant activity, as their numbers of fluorescent dots did not increase compared with the model group. Additionally, deaths were observed after exposure to 80 μg/mL of HD lipids, indicating potential cytotoxicity, which was also observed in the antithrombotic activity experiment.

## 4. Discussion

Seafood shellfish have tender and delicious meat, are rich in nutrients, and are widely loved by consumers. However, fresh shellfish are rich in moisture and highly perishable, making them unsuitable for long-term storage and transportation [23]. As a result, they are typically processed into dried products. Dried shellfish, such as razor clams, are widely consumed due to their minimal nutrient loss, favorable flavor, and texture [24]. This highlights the potential of dried shellfish products as a valuable source of nutrition and a marketable product. Nevertheless, there is currently no comprehensive research on the lipid composition and bioactivities of razor clams after different drying methods. In this study, we conducted a detailed analysis and comparison of the lipid composition of razor clams in the FD, HD, and FS groups using UPLC-MS/MS-based lipidomics. Additionally, we characterized the antithrombotic, anti-inflammatory, and antioxidant activities of the FD, HD, and FS lipids using zebrafish models for the first time. Our findings provide a foundation for the development and utilization of dried razor clam products in lipid studies.

Lipidomics is a discipline that systematically studies all lipids within cells, tissues, or living organisms. This is usually achieved through the analysis of mass spectrometry data, which can determine the structure and composition of lipid molecules. Additionally, changes in lipid composition and expression during various biological processes can be analyzed in detail [25,26,27]. In the present study, the lipidomic results showed that a total of 1056 lipid species were detected in the razor clams. GPs were the most predominant species, accounting for 57.39% of the total lipids, and razor clam lipids also contained a high proportion of PUFAs (51.33%). Both proportions were similar to those of the previous study [22]. The most abundant lipid subclasses were TG (26.14%), PE (16.10%), and PC (15.63%), respectively (Figure 1B). PC is critical for cell structure and function, and has been documented to be present in animal cells widely [28]. It has been demonstrated that PC exerts a pronounced effect in the management of inflammatory bowel disease [29]. TGs were closely related to cardiovascular disease and were also a reliable predictor of non-alcoholic fatty liver disease [30]. Meanwhile, as one of the most abundant phospholipids in mammalian plasma membranes, PEs can serve as a biomarker for cell death during disease processes [31,32].

According to statistical analysis, significant differences were observed in the contents of some lipid molecules among the FD, HD, and FS sample groups. The total number of high-level differential lipids was much greater than that of low-level lipids. And, the main differences were concentrated in PEs, PCs, LPEs, LPCs, TGs, and Cers. These differential lipids suggested that different processing methods (FD and HD) can have distinct effects on the lipid composition of razor clams. These lipid differences, in turn, affect the bioactivities and flavor of the products [33,34].

Currently, it has been reported that bivalve mollusks possess antimicrobial, antiviral, and antioxidant activities [35,36,37,38], and relevant analyses have also been conducted on the lipid composition of some species, such as *Ruditapes philippinarum* and *Sinonovacula constricta* [39,40]. However, there is limited research on the lipid activities of razor clams. Here, using an in vivo zebrafish model, we found the FD and FS lipids of razor clams exhibited antithrombotic, anti-inflammatory, and antioxidant activities at some concentrations, while HD lipids showed antithrombotic and anti-inflammatory activities but lacked antioxidant activity. The results indicate that the drying process has certain impacts on the activities of shellfish lipids, with FD having a smaller impact than HD, which may be due to the oxidation or decomposition of some lipids during the HD process [22]. And, the differential lipids between different lipid groups may contribute to the differences in activities.

In addition, the HD lipids at a concentration of 80 μg/mL caused deformities or death in zebrafish during the antithrombotic and antioxidant activity tests. The HD process may produce compounds that are harmful to the growth and development of zebrafish. Moreover, the lipids of razor clams are susceptible to oxidation during the drying process due to the high content of PUFAs. Oxidative stability is also influenced by the source, chemical composition, and quality of lipids [20]. Therefore, special attention should be paid to the selection of drying methods for clams. Further research is needed on the processing and development of shellfish lipids in the future.

## 5. Conclusions

Currently, research on razor clams mainly focuses on lipid composition and reaction mechanisms, with relatively little investigation into changes in lipid composition and bioactivity after the drying process. In this study, a total of 1056 lipid species were detected in razor clams, with GPs being major components, and TG, PE, and PC as the most abundant subclasses. Significant differences in lipids were concentrated in PEs, PCs, LPEs, LPCs, TGs, and Cers among the FD, HD, and FS sample groups. Additionally, the FD and FS lipids showed antithrombotic, anti-inflammatory, and antioxidant activities, while HD lipids only had antithrombotic and anti-inflammatory activities and exhibited toxicity at the concentration of 80 μg/mL. The results indicate that the lipid composition, bioactivity, and potential toxicity of clams are influenced by the drying method used. Compared with HD clams, FD clams have higher levels of PUFAs in their lipids, better bioactivity, and lower potential toxicity, making FD a more suitable method for clam drying. This is the first analysis and comparative study on the lipid composition and activity of razor clams after different drying methods and highlights the importance of considering different drying methods in the development of dried clam products. Furthermore, our research provides a reference for selecting drying methods for shellfish products and offers insight for the future development of clam lipids for nutritional and health products. However, there are still some shortcomings that can be improved in this study. On the one hand, only two methods, freeze drying and hot air drying, were studied, while other drying methods, such as spray drying, were not covered, which limits the generalizability of the conclusions. On the other hand, the bioactivity was only evaluated through zebrafish models, which may lead to incomprehensive research on activity, resulting in incomplete correspondence between activity results and actual effects in humans. In the future, more drying methods and bioactive models will be applied to the study of clam lipids.

## Data Availability

The original contributions presented in this study are included in the article/Supplementary Material. Further inquiries can be directed to the corresponding authors.

## Supplementary Materials

The following supporting information can be downloaded at: https://www.mdpi.com/article/10.3390/foods14060915/s1, Figure S1. Multivariate statistical analysis. (A) Principal component analysis (PCA) diagram for QC samples; (B-D) principal component analysis diagram between groups; (E) Partial Least Squares-Discriminant Analysis (PLS-DA) diagram; (F) permutation diagram. Figure S2. Volcano plot. Red dots represent significantly up-regulated differentiated lipids in the experimental groups, blue dots represent significantly down-regulated differentiated lipids, and grey dots represent lipids with no significant difference. Each point in the graph represents one lipid, the horizontal coordinate is the log_2_ (FC) value for the comparison of the two groups, and the vertical coordinate is the -log10 (*p*-value) value, with the red dots representing significantly up-regulated differentiated lipids (*p* < 0.05, VIP > 1, FC > 1) and the blue dots representing significantly down-regulated differentiated lipids (*p* < 0.05, VIP > 1, FC < 1). Figure S3. Lollipop map. Graphs show log_2_ (FC) in the horizontal coordinates, differential lipids in the vertical coordinates, red indicates up-regulation, blue indicates down-regulation, and asterisks indicate the significance of differential metabolism (* <0.05; ** <0.01; *** <0.001; **** <0.0001), with the size of the dots being determined by the VIP value. Table S1. Lipid molecular species in Sinonovacula constricta identified by UPLC-MS/MS method. Table S2. Differential lipids between FD and FS groups. Table S3. Differential lipids between HD and FS groups. Table S4. Differential lipids between FD and HD groups.
