# Supplementary material for: Effect of Freeze Drying and Hot Air Drying on the Composition and Bioactivities of Lipids from Razor Clam Sinonovacula constricta
Source: Foods. 2025 Mar 7;14(6):915. doi: 10.3390/foods14060915 (PMC11941059; doi:10.3390/foods14060915)
Supplement: Supplementary file 1 [file foods-14-00915-s001.zip › foods-3427606-supplementary.pdf]

Effect of freeze drying and hot air drying on the composition and bioactivities of lipids from razor clam *Sinonovacula constricta*

Dexu Wang [1,†], Runjia Chang [2,†], Changyu Liu [1], Jiaxun Li [1], Jibin Liu [1], Ning Li [1], Yun Zhang [1], Xiaobin Li [1], Peihai Li [1,*], Kechun Liu [1,*]

[1] Engineering Research Center of Zebrafish Models for Human Diseases and Drug Screening of Shandong Province, Biology Institute, Qilu University of Technology (Shandong Academy of Sciences), Jinan 250103, China; lining@sdas.org (N. Li)

[2] School of Life Sciences and Technology, Tongji University, Shanghai 200092, China; 2152673@tongji.edu.cn (R. Chang)

* Correspondence: E-mails: liph@sdas.org (P. Li), liukechun2000@163.com (K. Liu)

† These authors contributed equally to this work.

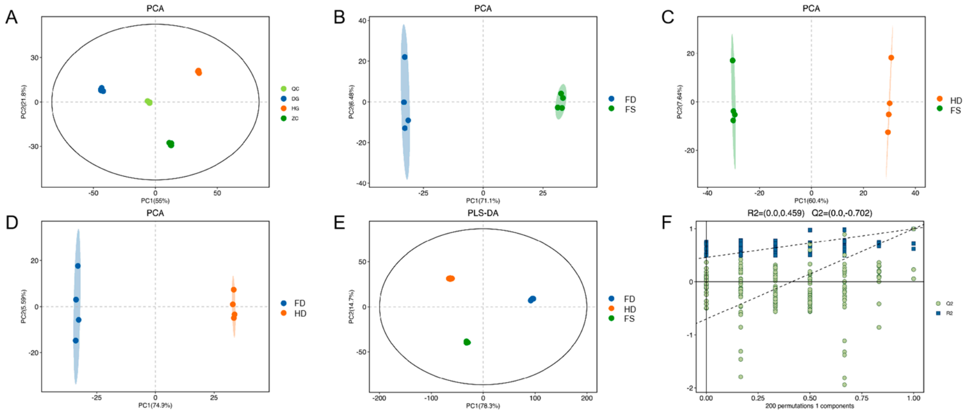

Figure S1. Multivariate statistical analysis. (A) Principal component analysis (PCA) diagram for QC samples; (B-D) principal component analysis diagram between groups; (E) Partial Least Squares-Discriminant Analysis (PLS-DA) diagram; (F) permutation diagram.

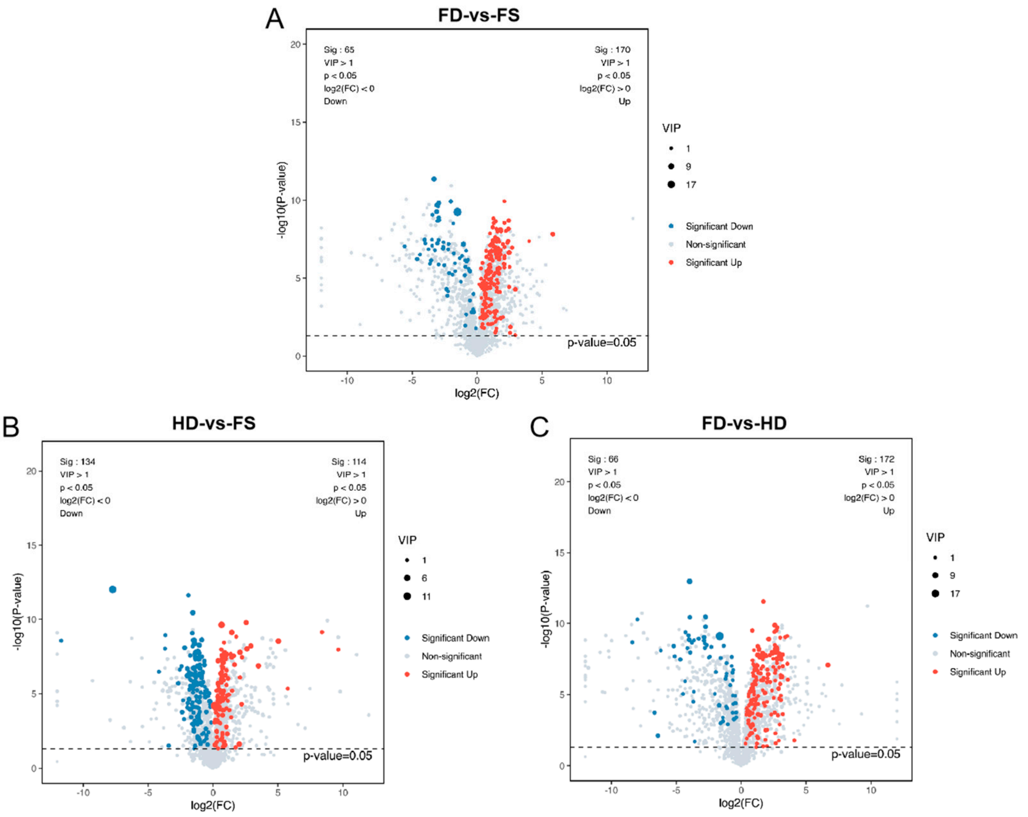

Figure S2. Volcano plot. Red dots represent significantly up-regulated differentiated lipids in the experimental groups, blue dots represent significantly down-regulated differentiated lipids, and grey dots represent lipids with no significant difference. Each point in the graph represents one lipid, the horizontal coordinate is the $\log_2$ (FC) value for the comparison of the two groups, and the vertical coordinate is the -log10 (P-value) value, with the red dots representing significantly up-regulated differentiated lipids (P<0.05, VIP>1, FC>1) and the blue dots representing significantly down-regulated differentiated lipids (P<0.05, VIP>1, FC<1).

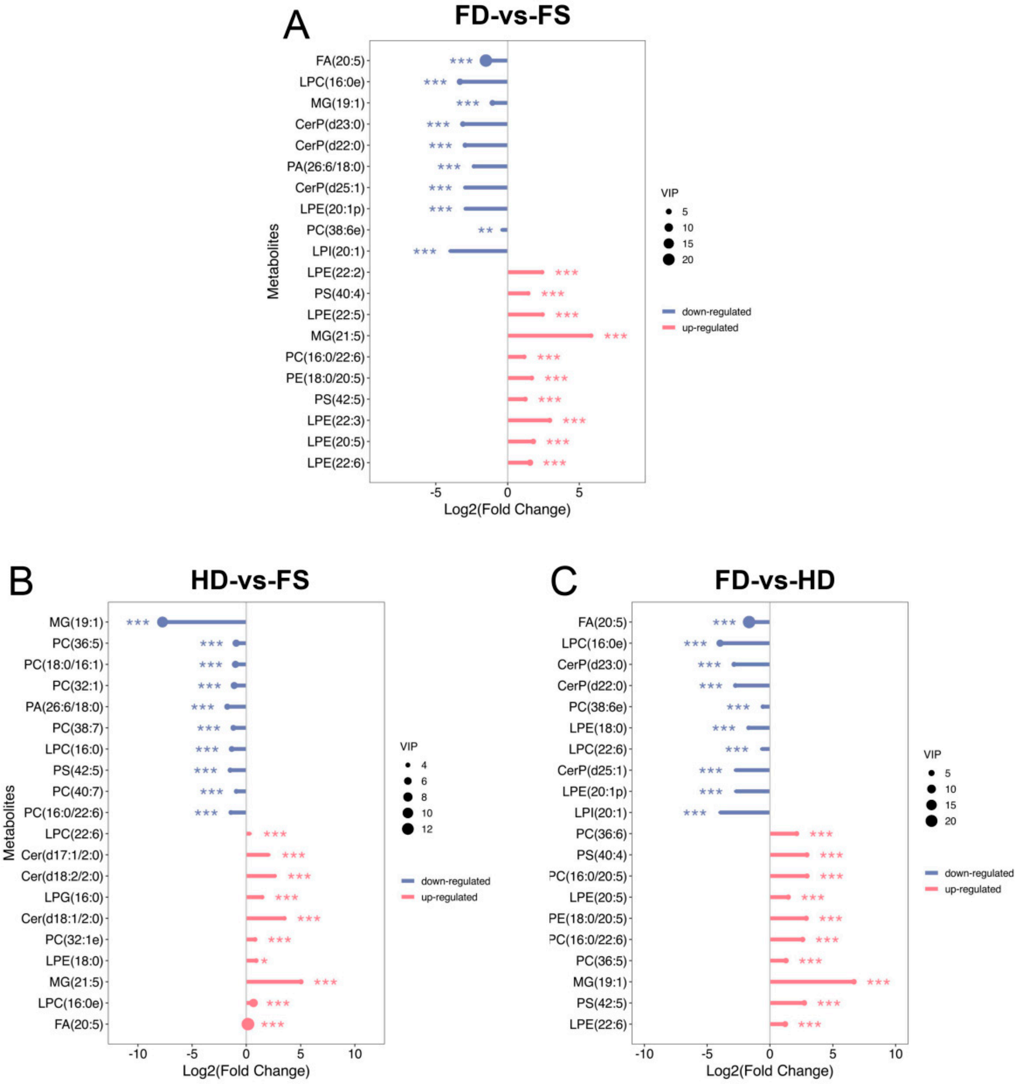

Figure S3. Lollipop map. Graphs show $\log_2$ (FC) in the horizontal coordinates, differential lipids in the vertical coordinates, red indicates up-regulation, blue indicates down-regulation, and asterisks indicate the significance of differential metabolism (*<0.05; **<0.01; ***<0.001; ****<0.0001), with the size of the dots being determined by the VIP value.

S1. Pretreatment of the samples

Each group of razor clam meat lipid extracts extracted by different methods was divided into four groups each, 30 mg of samples were weighed in each group, 600 μL of isopropanol-methanol (V:V=1:1) was added, and 20 μL of a mixed internal standard (4 μg/mL, methanol configuration) was added. Two small steel balls were added and placed at -20°C for 2 min to pre-cool, and were then put into a grinder (60 Hz, 2 min). The sample was extracted by ultrasonication for 10 min, allowed to stand at -20°C for 20 min, and centrifuged for 10 min (13000 rpm, 4°C). An amount of 150 μL of the supernatant was loaded into LC-MS injection vials with liner tubes and used for LC-MS analysis. Quality control (QC) samples were prepared by mixing equal volumes of extraction reagents from all samples, and the volume of each QC was the same as that of the sample. All extraction reagents were pre-cooled at -20°C before use.

S2. Database

The LipidSearch Lipidome Database is a lipidomics database based on the QE series of high-resolution mass spectrometry instruments introduced by Thermo Scientific. It is regarded as one of the most prominent databases for lipidomics research. The database contains a comprehensive collection of lipid molecules, encompassing over 8 major classes, 300 subclasses, and 1.7 million lipid molecules along with their predicted fragmentation ion spectra. These are then analyzed by various lipid characterization algorithms, including sub-ion, precursor and neutral loss scans. This multifaceted approach ensures systematic and reliable lipid characterization. The database's capabilities extend to qualitative analysis through the utilization of advanced lipid identification algorithms, such as daughter ion, precursor ion, and neutral loss scanning.

S3. Qualitative analysis

Utilizing Lipid Search software, the raw data in a raw format exported by Q Exactive LC-MS/MS are read, and the exact mass numbers of MSn and parent ions are identified. The structure of the lipid molecules and their positive and negative ion addition patterns are identified based on the parent ions and multistage mass spectrometry data in each individual sample. For each independent sample, the search results were then aligned according to a specific retention time timeframe, and the results were combined into a single report, collating the raw data matrix.

S4. Qualitative quantitative results

In each sample, the total peak area was normalized for all peak signal intensities (peak areas). That is to say, each peak signal intensity was used to convert to a relative intensity in that spectrogram. Following this, the data were multiplied by 10,000. For the extracted data, ion peaks with >50% missing values (0 values) were deleted for each group; the remaining missing values (0 values) were replaced with half of the minimum value. (See the missing value data matrix in the Data Matrix for data without 0-value replacement.)
